# Coding-Complete Sequences of Recombinant Lumpy Skin Disease Viruses Collected in 2020 from Four Outbreaks in Northern Vietnam

**DOI:** 10.1128/MRA.00897-21

**Published:** 2021-12-02

**Authors:** Elisabeth Mathijs, Frank Vandenbussche, Long Nguyen, Laetitia Aerts, Tho Nguyen, Ilse De Leeuw, Minh Quang, Hoang Dang Nguyen, Wannes Philips, Thi Vui Dam, Andy Haegeman, Steven Van Borm, Kris De Clercq

**Affiliations:** a Sciensano, Exotic Viruses and Particular Diseases Unit, Ukkel, Belgium; b Department of Animal Health (DAH), Ministry of Agriculture and Rural Development, Phuong Dinh, Dong Da, Hanoi, Vietnam; c National Centre For Veterinary Diagnostics (NCVD), Phuong Mai, Dong Da, Hanoi, Vietnam; Queens College CUNY

## Abstract

*Lumpy skin disease virus* (LSDV) causes a severe, systemic, and economically important disease in cattle. Here, we report coding-complete sequences of recombinant LSDVs from four outbreaks in October and November 2020 in northeastern Vietnam.

## ANNOUNCEMENT

L umpy skin disease (LSD) is a viral disease in cattle with important economic losses. The disease is caused by the lumpy skin disease virus (LSDV), a double-stranded DNA virus belonging to the genus *Capripoxvirus* (CaPV) in the family *Poxviridae*. Recently, LSD began spreading in the eastern part of the Russian Federation, China, and Southeast Asia. On 1 November 2020, Vietnam reported its first outbreak of LSDV in cattle in the region of the northeastern border with China (1). The disease quickly spread across the entire country. We obtained near-complete genome sequences of LSDVs from four of the first outbreaks in northeastern Vietnam: 20L42_Quyet Thang/VNM/20 (Quyết Thằng, Hữu Lũng District), 20L43_Ly Quoc/VNM/20 (Lý Quô’c, Hữu Lũng District), 20L70_Dinh To/VNM/20 (Đình Tô', Thuâ.n Thành District), and 20L81_Bang Thanh/VNM/20 (Bằng Thành, Pằc Nă.m District).

DNA was purified from skin samples collected for LSDV diagnosis using the Puregene Core kit A (Qiagen) as previously described (2). Twenty-three overlapping PCR products (ranging between 7,417 and 7,852 bp) covering the entire genome were amplified using Q5 high-fidelity DNA polymerase (New England Biolabs) (E. Mathijs, A. Haegeman, K. De Clercq, S. Van Borm, F. Vandenbussche, submitted for publication). To distinguish between the inverted terminal repeats (ITR), two libraries, each compromising a pool of PCR amplicons corresponding to half of the CaPV genome, were prepared using the Nextera XT library preparation kit (Illumina). MiSeq sequencing (reagent kit v3 with 2 × 300-bp paired-end sequencing; Illumina) was performed. Information about the data generated for all four samples is given in Table 1. Trim Galore v0.3.8 (http://www.bioinformatics.babraham.ac.uk/) was used for read trimming based on quality (Q score, >30) and length (>80 bp; 5′ clip for R1 and R2, 20). For each library, a subset of 20,000 trimmed paired-end reads (theoretical coverage, 50×) were assembled *de novo* into a single contig using SPAdes v3.9.0 with k values of 21, 33, and 55 (3). No nucleotide variants were identified using the LoFreq v2.1.3.1 variant caller (4). Default parameters were used for all software unless otherwise specified. The contigs from both libraries were manually merged into a single sequence of at least 150,551 bp, with an evenly distributed average GC content of 25.93% and an average coverage depth of minimum 2,537× (Table 1). All four sequences are characterized by a 145,885-bp central coding region, flanked by two ITRs of at least 2,164 bp, and contain all expected LSDV open reading frames (ORFs). With the exception of a single nucleotide mutation in LSDV073 (S26L) for 20L43_Ly Quoc/VNM/20, all four coding genome sequences were identical at the nucleotide level. NCBI BLAST analysis (5) showed that the Vietnamese field strains share 99.99% and 99.41% nucleotide identity with the LSDV field isolates China/GD01/2020 (GenBank accession no. MW355944) and Russia/Saratov/2017 (MH646674), respectively. Annotation and amino-acid gene prediction was performed using GATU software (downloaded from https://4virology.net/virology-ca-tools/gatu/; accessed 24 Feb 2020) (6) relative to the LSDV field isolate China/GD01/2020 (MW355944). The LSDV strains characterized from these first Vietnamese outbreaks are most closely related to contemporary recombinant LSDV strains from China and Russia (7, 8). These strains are in fact patchwork genomes resulting from multiple recombination events involving a least one field strain and one vaccine LSDV strain. This finding highlights the importance of complete genomes in LSDV outbreak tracing.

**TABLE 1:** Summary of the sequencing and assembly results of the 20L42_Quyet Thang/VNM/20, 20L43_Ly Quoc/VNM/20, 20L70_Dinh To/VNM/20, and 20L81_Bang Thanh/VNM/20 data sets

Sample ID	Library	No. of reads	Contig length (bp)	%GC	Mean coverage depth (×)	GenBank accession no.
20L42_Quyet Thang	1	438,944	150,665	25.93	2,945	MZ577073.1
	2	447,798				
20L43_Ly Quoc	1	585,237	150,599	25.93	4,260	MZ577074.1
	2	605,618				
20L70_Dinh To	1	431,910	150,600	25.93	2,807	MZ577075.1
	2	431,020				
20L81_Bang Thanh	1	508,084	150,664	25.93	2,537	MZ577076.1
	2	433,720				

### Data availability.

The LSDV sequences from this study have been deposited in GenBank under accession numbers MZ577073.1 to MZ577076.1, and the raw data have been submitted to the SRA under BioProject accession number PRJNA746718.
